# Efficient Non-Viral Reprogramming of Myoblasts to Stemness with a Single Small Molecule to Generate Cardiac Progenitor Cells

**DOI:** 10.1371/journal.pone.0023667

**Published:** 2011-08-17

**Authors:** Zeeshan Pasha, Husnain Kh Haider, Muhammad Ashraf

**Affiliations:** Department of Pathology, University of Cincinnati, Cincinnati, Ohio, United States of America; Brigham and Women's Hospital, United States of America

## Abstract

The current protocols for generation of induced pluripotent stem (iPS) cells involve genome integrating viral vectors which may induce tumorgenesis. The aim of this study was to develop and optimize a non-viral method without genetic manipulation for reprogramming of skeletal myoblasts (SMs) using small molecules.

**Methods and Results:**

SMs from young male Oct3/4-GFP^+^ transgenic mouse were treated with DNA methyltransferase (DNMT) inhibitor, RG108. Two weeks later, GFP^+^ colonies of SM derived iPS cells (SiPS) expressing GFP and with morphological similarity of mouse embryonic stem (ESCs) were formed and propagated i*n vitro*. SiPS were positive for alkaline phosphatase activity, expressed SSEA1, displayed ES cell specific pluripotency markers and formed teratoma in nude mice. Optimization of culture conditions for embryoid body (EBs) formation yielded spontaneously contracting EBs having morphological, molecular, and ultra-structural similarities with cardiomyocytes and expressed early and late cardiac markers. miR profiling showed abrogation of let-7 family and upregulation of ESCs specific miR-290-295 cluster thus indicating that SiPS were similar to ESCs in miR profile. Four weeks after transplantation into the immunocompetent mice model of acute myocardial infarction (n = 12 per group), extensive myogenesis was observed in SiPS transplanted hearts as compared to DMEM controls (n = 6 per group). A significant reduction in fibrosis and improvement in global heart function in the hearts transplanted with SiPS derived cardiac progenitor cells were observed.

**Conclusions:**

Reprogramming of SMs by DNMT inhibitor is a simple, reproducible and efficient technique more likely to generate transgene integration-free iPS cells. Cardiac progenitors derived from iPS cells propagated extensively in the infarcted myocardium without tumorgenesis and improved cardiac function.

## Introduction

The introduction of four transcription factors, Oct3/4, Sox2, Klf4, and c-Myc can successfully reprogram mouse and human somatic cells into induced pluripotent stem (iPS) cells [1], [2]. However the current reprogramming approach requires viral transduction which hampered the use of iPS due to genetic variability caused by random integration of multiple pro-viral copies [3], [4]. Several multidisciplinary approaches involving the use of non integrating adenoviral vectors [5], non-viral plasmid transfection [6], [7] and piggy Bac transposition [8] had been attempted by various research groups to refine and improve the iPS cell generation. Given that Oct3/4, Sox2, and Nanog constitute the core of regulatory loop which is imperative to determine the pluripotent status of the cells, other studies showed the dispensable status of cMyc and Klf4 for reprogramming process [9], [4]. However, Oct3/4 apparently cannot be replaced for pluripotency by other factors. Oct3/4 is a key regulator of mouse embryogenesis and is expressed in the pluripotent cells of an embryo and in cell lines derived thereafter [10]. Given the molecular pathways underlying iPS cell generation, the use of lesser number of transcription factors combined with small molecules is being promoted for pluripotency [9], [11]. For epigenetic reprogramming, inhibition of DNMT and histone deacetylases with small molecules is important to reduce transcription of silenced genes [12], [13]. Therefore, the possibility to reactivate epigenetically silenced genes has generated considerable interest in the use of DNMT inhibitors. RG108, a DNMT inhibitor, is a potent small molecule that effectively blocks DNMT *in vitro* and does not cause covalent enzyme trapping in human cell lines [14]. We report here for the first time that mouse skeletal myoblasts (SMs) can be efficiently reprogrammed into iPS cells (SiPs) with DNMT inhibitor by induction of a single transcription factor Oct3/4. These SiPS cells resemble ES cells in their molecular behavior and differentiation characteristics. We further report that cardiac progenitors (SiPS-CPs) derived from beating EBs obtained from SiPS showed remarkable regeneration of myocardium and formed gap junctions with the resident cardiomyocytes when transplanted in an infarcted mouse heart. We also observed a significant attenuation of infarct size expansion and concomitantly improved global heart function in SiPS-CPs transplanted animal hearts. Our purely chemical approach is superior and safest in efficient reprogramming of SMs for generation of cardiac progenitors.

## Materials and Methods

### Isolation of mouse SMs

For our animal experiments, we used the Oct4/GFP transgenic mouse strain (Jackson laboratories, Maine, USA) with GFP-tagged to the endogenous Oct3/4 gene promoter. For SMs isolation, we followed the standard protocols routinely used in our laboratory as described in Text S1.

### SiPS generation and maintenance

SMs derived from Oct3/4-GFP mice (at passage 1–2; 1×10^5^ cells/well of a 6-well dish) were treated overnight with 500 µM RG108 (Stemgent, CA, USA) in 0.5% DMSO for 5 days. Control cells were treated with DMSO 0.05% without RG108. At day 6, the treated cells were passaged on the mouse embryonic fibroblasts (MEF) coated 10 cm cell culture dishes and observed for the development of SiPS clones until 3 weeks. The cell growth media was changed daily. On day 15, appearance of ES cells like GFP^+^ clones were observed and counted. The GFP^+^ SiPS clones were mechanically incised, cultured on mouse feeder cells and expanded individually in ES cell culture medium for use in further experiments. For induction of pluripotency markers, SiPS were fixed with 4% paraformaldehyde, permeabilized and stained with anti-stage specific embryonic antigen-1 (SSEA-1) antibody. The primary antigen-antibody reaction was detected with goat anti-mouse Alexa Fluor-568 conjugated secondary antibody (1∶ 200; Cell Signaling Tech, Danvers, MA). Nuclei were visualized by 4,6′ -diamidino-2-phenylindole (DAPI; Invitrogen, Carlsbad, CA) staining. The murine SiPS clone ZP1 was expanded on mitotically inactivated murine embryonic fibroblasts (MEFs; 5×10^4^cells/cm^2^) and maintained as described in Text S1.

### Reverse transcription polymerase chain reaction (RT-PCR)

Isolation of total RNA, and their subsequent first-strand cDNA synthesis, was performed using an RNeasy mini kit (Qiagen, Valencia, CA) and an Omniscript Reverse Transcription kit (Qiagen, Valencia, CA) respectively per manufacturer's instructions and detailed in Text S1. The primer sequences used are given in Table S1.

### Alkaline phosphatase staining and immunocytochemistry

Alkaline phosphatase staining was performed using Alkaline Phosphatase Detection kit (Millipore SCR2004) per manufacturer's instructions. For immunocytochemistry, undifferentiated colonies of SiPs were immunostained with respective specific primary antibodies (anti-SSEA1, anti-Oct3/4, anti-Sox2 antibodies, all at 1∶ 100 dilutions; Cell Signaling, Danvers, USA) as described in Text S1. Fluorescence signals were observed and photographed using fluorescence microscope (Olympus, Tokyo, Japan).

### DNA methyltransferase (DNMT) activity assay

Nuclear extracts were isolated using the NE-PER Nuclear and Cytoplasmic Extraction Kit (Thermo Scientific, IL USA). Total DNMT activity was determined using an EpiQuik DNA methyltransferase activity assay kit (Epigentek, Brooklyn, NY) per manufacturer's protocol. Enzyme activity for samples and controls was measured on a microplate reader (Hidex Chameleon, Finland) at 450 nm and DNMT activity (OD/h/ml) was calculated according to the formula: (Sample OD−blank OD)/(sample volume)×1000.

### Embryoid body formation for spontaneous cardiac differentiation

SiPS were cultured in ultralow attachment dishes (Corning, NY, USA) for 3 days in high glucose DMEM supplemented with 15% FBS, 0.1 mmol/L non-essential amino acids, 1 mmol/L L-glutamine, 0.1 mmol/L β-mercaptoethanol, and 5 mM Pencillin/Streptomycin. After 3 days in cell suspension, rounded EBs were formed that were seeded on gelatin-coated dishes and cultured for 15 days. The contracting EBs on day 5 were microdissected and dissociated into single cells for further experiments including immunostaining and RT-PCR.

### Ultra-structural studies of EBs derived cardiac progenitors

Ultra-structural studies were performed on SiPS-CP derived from 5-day beating EBs as described in Text S1 using JEOL transmission electron microscope.

### Teratoma formation and Karyotyping

Teratogenicity of SiPS was assessed in immunodeficient mice (n = 3) as described in Text S1. Karyotyping was determined at molecular cytogenetic facility of Ohio State University, OH.

### MicroRNA microarray Analysis

A total of 4–8 µg RNA sample each was prepared from SMs, mouse ES cells and SiPs. MicroRNA expression profiling was performed using a microarray service provider (LC Sciences, TX, USA) and as described in Text S1.

### Experimental animal model of myocardial infarction

All experimental procedures were performed in accordance with the standard human care guidelines of the “Guide for the Care and Use of Laboratory Animals” and were approved by the Institutional Animal Care and Use Committee of University of Cincinnati (Protocol ID: 06-03-13-03), which conforms to National Institutes of Health guidelines. Myocardial Infarction model was induced in young female 8–12 weeks old immunocompetent C57BL/6J mice [15]. Briefly, the animals were anesthetized with (ketamine/xylazine 0.05 ml intra-peritonealy). A midline cervical skin incision was performed for intubation. The animals were mechanically ventilated with room air supplemented with oxygen (1.5 L/min) using a rodent ventilator (Model 683, Harvard Apparatus, MA, USA). Body temperature was carefully monitored with a probe (Cole-Parmer Instrument, IL, USA) and was maintained at 37°C throughout the surgical procedure. The heart was exposed by left-sided limited thoracotomy and the left anterior descending (LAD) coronary artery was ligated with a prolene #9-0 suture. Myocardial ischemia was confirmed by color change of the left ventricular wall. The animals were grouped (n = 12 per group) for intramyocardial injection of 10 µL of basal DMEM without cells (group-1) or containing 3×10^5^ SiPS (group-2) or 3×10^5^ SiPS-CPs (group-3). The cells were injected 10 minutes after coronary artery ligation at multiple sites (3–4 sites per heart) in the free wall of the left ventricle under direct vision. For post-engraftment tracking of the transplanted cells and determination of their fate, the cells were labeled with PKH26 (Sigma, Product# PKH26-GL) according to manufacturer's instructions. The chest was closed and the animals were allowed to recover. To alleviate pain, Buprinex (0.05 ml) was injected subcutaneously in first 24 hours of surgery. The animals were euthanized on 7 days and 4 weeks after transthoracic echocardiography (as detailed in Text S1) for the heart function evaluation. The hearts were frozen or fixed with 10% formalin solution and processed for embedding in paraffin for immunohistological studies.

### Transthoracic Echocardiography

The animals (n = 8 per group) were anesthetized and lightly secured in the supine position on a warm pad. After the chest was shaven, Acoustic gel was applied and transthoracic echocardiography was performed using HDI-5000 SONOS-CT (HP) ultrasound machine with a 7-MHz transducer. The heart was imaged in the two-dimensional mode in the parasternal long-axis and/or parasternal short-axis views which were subsequently used to position the M-mode cursor perpendicular to the ventricular septum and left ventricle posterior wall, after which M-mode images were obtained. For each animal, measurements were obtained from 4–5 consecutive heart cycles. Measurements of ventricular septal thickness (VST), left ventricle internal dimension (LVID), and left ventricle posterior wall thickness (LVPW) were made from two-dimensionally directed M-mode images of the left ventricle in both systole and diastole. The average value from all measurements in an animal were used to determine the indices of left ventricle contractile function, i.e., left ventricle fractional shortening (LVFS) and left ventricle ejection fraction (LVEF) using the following relations LVFS = (LVEDd−LVESd)/LVEDd×100 and LVEF = [(LVEDd^3^−LVESd^3^)/LVEDd^3^]×100 and expressed as percentages.

## Results

### Reprogramming of SMs with DNMT inhibitor

**Figure 1:**
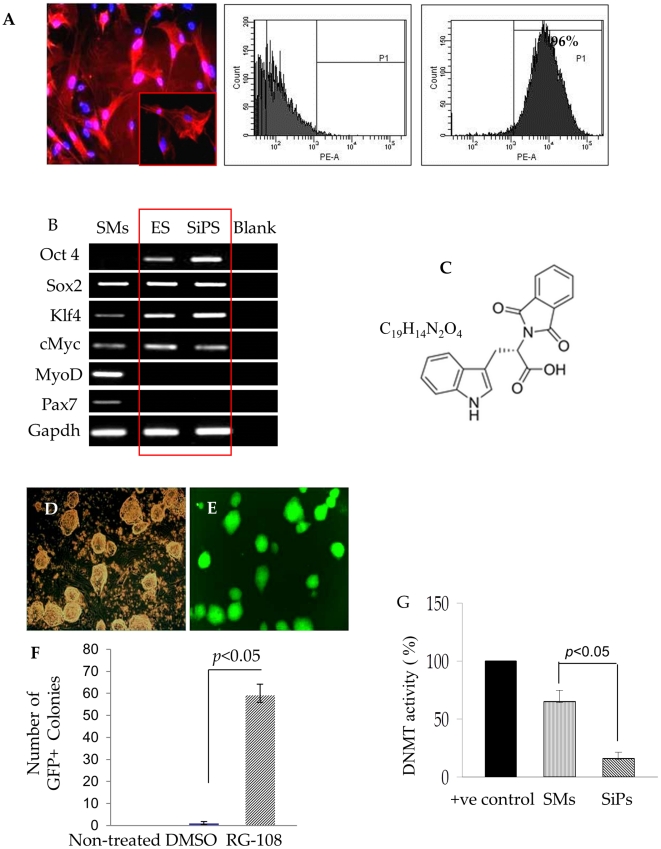
SiPS generation from SMs by single small molecule treatment. (A) The native SMs cells were stained for desmin expression (red fluorescence) and confirmed by flow cytometry (96%) to show the purity of SMs. The nuclei were stained with DAPI (blue fluorescence). (B) Gene expression profile of various stemness and myogenic markers in SMs, ES cells and SiPS isolates. RT-PCR analyses showing the endogenous expression of myogenic and pluripotency marker genes in SMs and ES cells. SMs showed 3 pluripotency markers (Sox2, Klf4, cMyc) in addition to MyoD and Pax7 in comparison to ES cell expressing all 4 pluripotency markers. RG108 significantly induced Oct3/4 expression in SiPS with simultaneous loss of myogenic markers MyoD and Pax7 after reprogramming. Densitometric quantization of mRNA expression of Oct3/4, Sox2, Klf4, cMyc, MyoD and Pax7 in SMs, ES cells and SiPS are given in Figure S1. (C) Chemical structure of RG108 (D) Phase contrast and (E) fluorescent images of GFP expressing SiPS clones generated by RG108 (500 µM). (F) Graph showing generation of approximately 57–60 GFP^+^ clones from 50,000 SMs 2–3 weeks after RG108 treatment. The control SMs were either without treatment or treated with DMSO (the solvent for RG108). Similar results were obtained in three independent experiments. (G) DNA methylation analysis showing significant inhibition of DNA methyltransferase activity in SiPS cells in comparison to the untreated SMs. Positive control shown in the graph was provided with the assay kit.

Mouse SMs were characterized for purity for desmin expression by desmin immunostaining and flow cytometry which revealed more than 96% purity of the cell culture (Figure 1A). The SMs endogenously expressed myogenic markers MyoD and Pax7 and 3 out of 4 pluripotency markers, Sox2, Klf4, cMyc as compared to ES cells (Figure 1B). SMs were treated with DNMT inhibitor, RG108 (Figure 1C). The successful reprogramming of SMs was evident from their ES cell-like morphology and Oct4 driven GFP expression (phase contrast and green fluorescence; Figure 1D–E). This treatment resulted in formation of 57 GFP^+^ SiPS colonies per 50×10^3^ cells within two weeks (Figure 1F). On the other hand, no colonies were formed in control SMs treated with DMSO (solvent for RG108) or without RG108 treatment. DNA methylation analysis showed that RG108 significantly reduced the DNMT activity in SiPS as compared to the native SMs. A positive control was provided with the kit (Figure 1G).

### Characterization of SiPS generated with DNMT inhibitor

**Figure 2:**
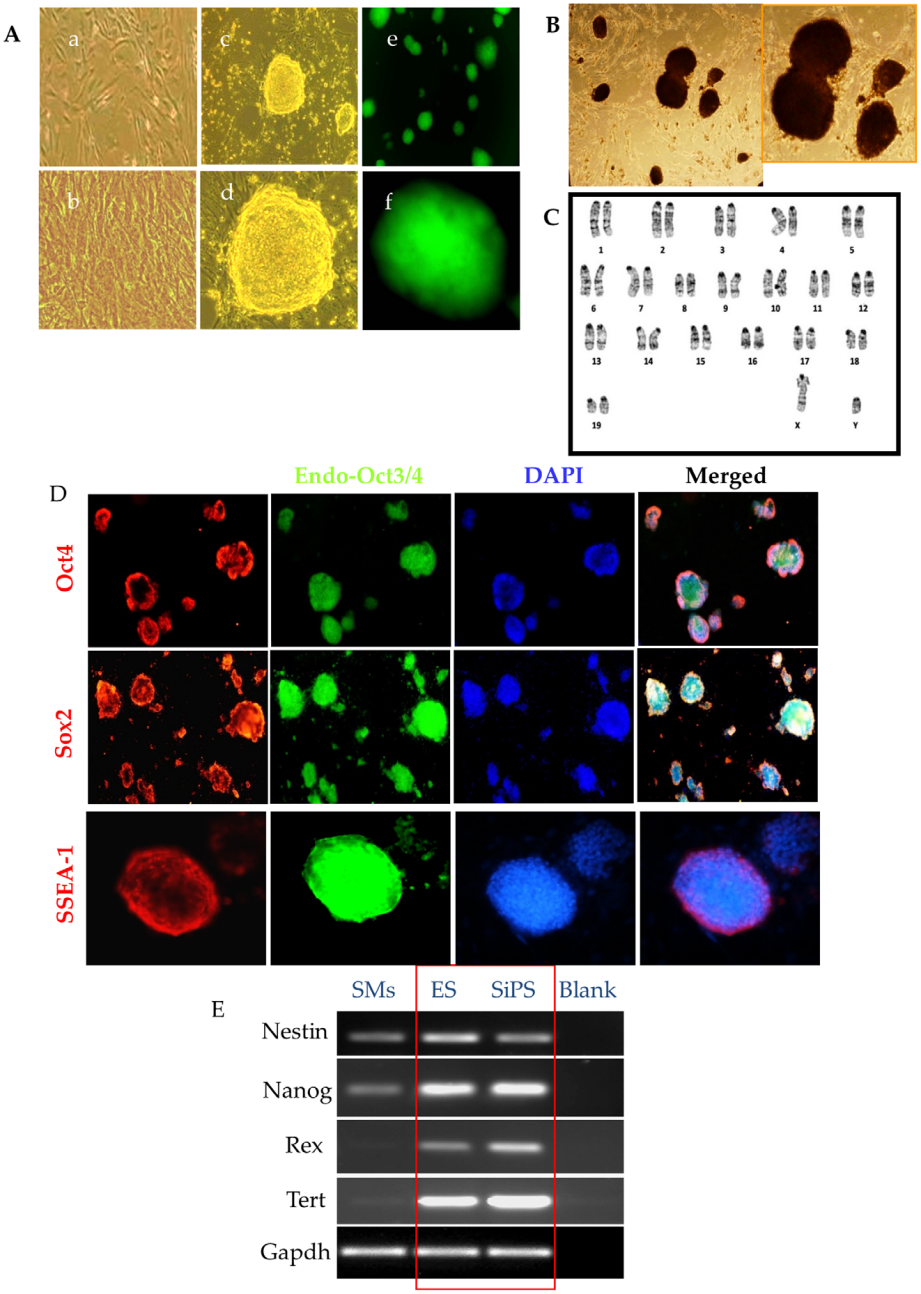
Characterization of SiPS for the expression of pluripotency markers. (A) Phase-contrast images of SiPS clones from RG108 treatment. Representative photomicrographs showing (a) untreated SMs on day 0 and (b) untreated SMs on day 15 showing fibroblast-like morphology (c–f) Phase-contrast and GFP fluorescent images (green fluorescence) of SiPS clones 15 days after RG108 treatment (B) SiPS clones stained for alkaline phosphatase activity. (C) SiPS clone showing normal karyotyping. (D) Immunostaining of SiPS ZP clone-1 expressing ES cell specific markers Oct3/4, Sox2 and SSEA-1 (red fluorescence) and endogenous Oct4 (green fluorescence). Nuclei were stained with DAPI (blue fluorescence). (E) RT-PCR analyses showing the endogenous gene expression of Nestin, Nanog, Rex-1, and Tert in SMs, ES and SiPS cells.

**Figure 3:**
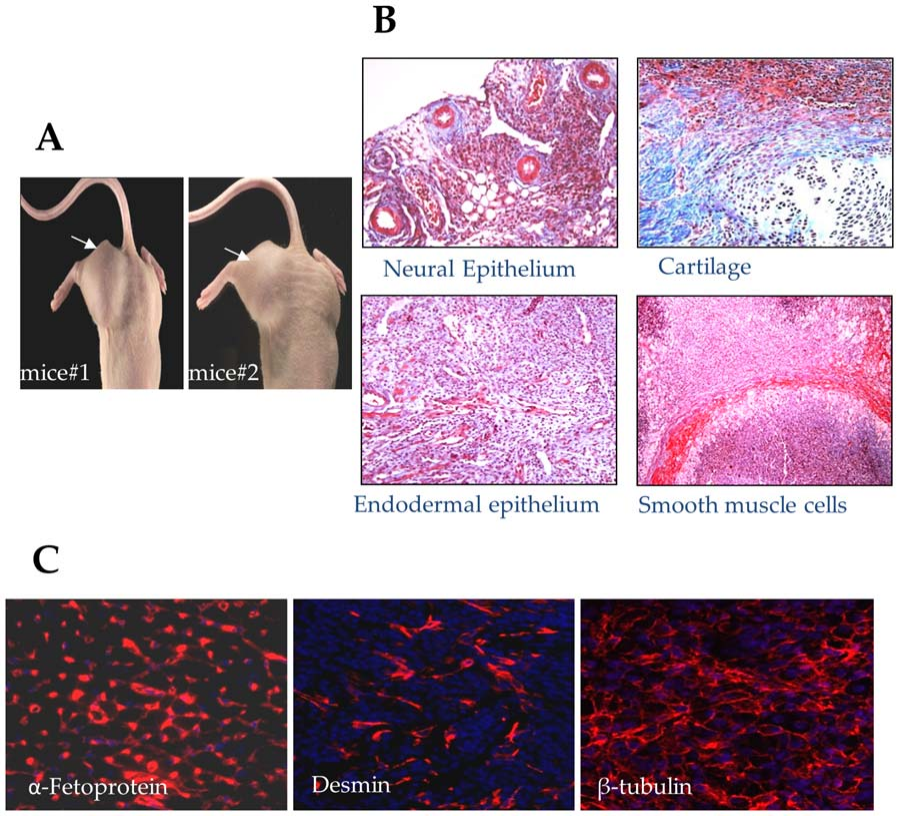
Teratoma formation by SiPS upon transplantation in an immunodeficient mice. (A) Teratoma formation in nude mice (n = 3) at 2 weeks after SiPS transplantation. (B) Histochemical staining for neural epithelium, cartilage, endodermal epithelium and smooth muscle (C) Immunohistochemical studies for α–fetoprotein, desmin, and β-tubulin, to characterize teratoma from nude mice for differentiation into three germ layers.

Culture of the undifferentiated SiPS on MEF or on gelatin coated dishes without feeder layers exhibited a typical ES-cell like morphology which appeared as compact, opaque, round clusters with well-defined margins in undifferentiated state and expressed Oct3/4 promoter driven GFP (Figure 2A). In comparison to SiPS, native SMs retained their morphological characteristics after 20 days as elongated spindle shape and didn't express Oct3/4 promoter driven GFP thus indicating their non-transformed status (Figure 2A, b). The undifferentiated SiPS were positive for alkaline phosphatase activity (Figure 2B) and had normal karyotypes (Figure 2C) for more than 25 passages. Fluorescence immunostaining showed that DNMT inhibitor successfully reprogrammed SMs to pluripotent status and induced ES cell specific markers including endogenous Oct3/4, Sox2, and SSEA-1 (Figure 2D). RT-PCR analysis confirmed these observations and showed that SiPS expressed pluripotency markers Oct3/4, Sox2, Klf4, and cMyc unlike native SMs which only expressed Sox2, Klf4, and cMyc markers (Figure 1B). Additionally, SiPS also expressed Nestin, Nanog, Rex1, Tert similar to ES cells (Figure 2E). These data indicated that SiPS have primitive gene expression profile similar to the ES cells. Subcutaneously injected SiPS formed teratomas in immunodeficient mice (n = 3) which showed typical three germ layer characteristics (Figure 3).

### *In vitro* differentiation of SiPS into spontaneously contracting cardiac progenitors

**Figure 4:**
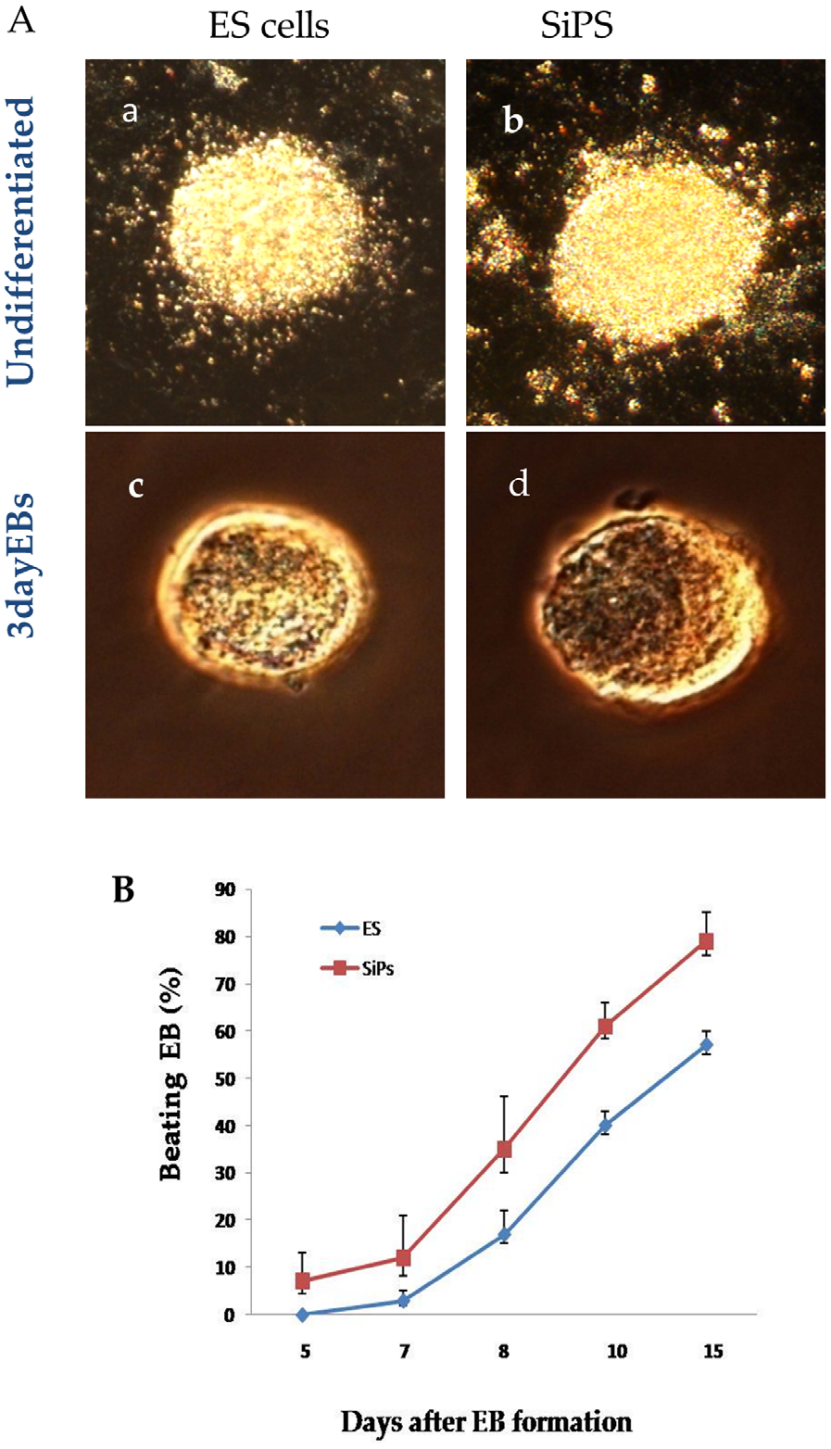
Embryoid bodies formation and beating pattern of murine ES cells and SiPS. (A) Phase contrast images showing the comparable growth characteristics of undifferentiated (a) ES cells and (b) SiPS. Three days old embryoid bodies (EBs) derived from (c) ES cells and (d) SiPS. (B) Spontaneously beating EBs from the ES and SiPS over time. Data are mean ±SEM.

The SiPS colonies derived from ZP clone-1 clone were propagated in an undifferentiated state on MEF feeder layer (Figure 4A). For *in vitro* cardiomyogenesis, SiPS cells were removed from MEF feeder and cultured in suspension, where these cells formed 3 dimensional differentiating cell aggregates (EBs) similar to ES cells (Figure 4A). After 3 days in suspension, EBs were seeded on gelatin-coated plates. Typical spontaneously contracting EBs derived SiPS-CPs first appeared as early as 5 days (Video S1) and the proportion of spontaneously contracting EBs increased up to 15% in 2 weeks (Figure 4B) and continued to beat robustly for 3–4 weeks in culture.

### Molecular characterization and immunostaining of SiPS derived cardiac progenitors

**Figure 5:**
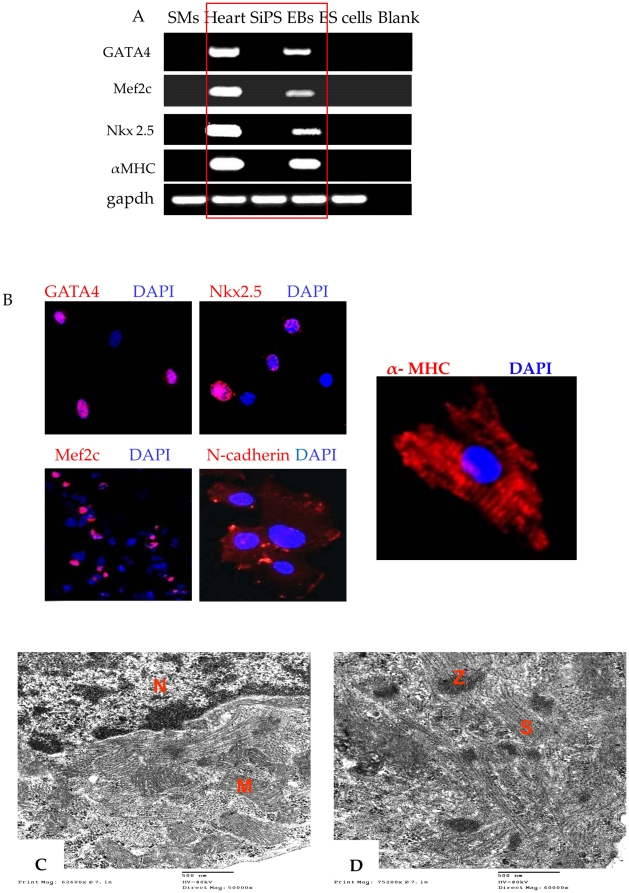
*In vitro* cardiomyogenic differentiation of SiPS. SiPS were cultured in suspension for 3 days culture. (A) RT-PCR analysis of 5 days old spontaneously beating EBs for cardiomyocyte specific markers, Gata4, Mef2c, Nkx2.5, and α-MHC., in comparison with SMs, undifferentiated SiPS and ES cells. Mouse heart was used as positive control. Quantitative mRNA expression of Gata4, Mef2c, Nkx2.5, and α-MHC in SMs, mouse heart, SiPS, 5 days EBs and ES cells are shown in Figure S2. (B) Immunostaining of cardiac specific genes in 5 days EBs cells which were positive for cardiac specific antigens Gata4, Nkx2.5 and Mef2c (merged images with DAPI). These cells were also positive for myosin heavy chain and expressed N-cadherin (red fluorescence). (C–D) Ultra-structural features of SiPS-CPs *in vitro*. Transmission electron micrographs of SiPS-CPs showing typical striated sarcomeres (s) with z-lines (z), nucleus (N) and mitochondria (M) (original magnifications; A = 50000×; B = 60000×).

We analyzed the 5 day beating EBs derived cardiac progenitors for cardiac specific markers in comparison to native SMs, undifferentiated SiPS and ES cells using mouse heart as a positive control. The expression of a series of cardiac marker genes including Gata4, Nkx2.5, Mef2c, and α-MHC was significantly increased at 5 days of differentiation (Figure 5A). Contrarily, no expression of these markers was observed in undifferentiated SiPS and ES cells. SiPS-CPs were positively immunostained for Gata4, Nkx2.5, Mef2c, N-cadherin and α-MHC (Figure 5B). Transmission electron microscopy revealed ultra-structural features of 5 days beating EBs with fully developed sarcomeres or myofilaments (Figure 5C–D).

### MicroRNA microarray analysis

**Figure 6:**
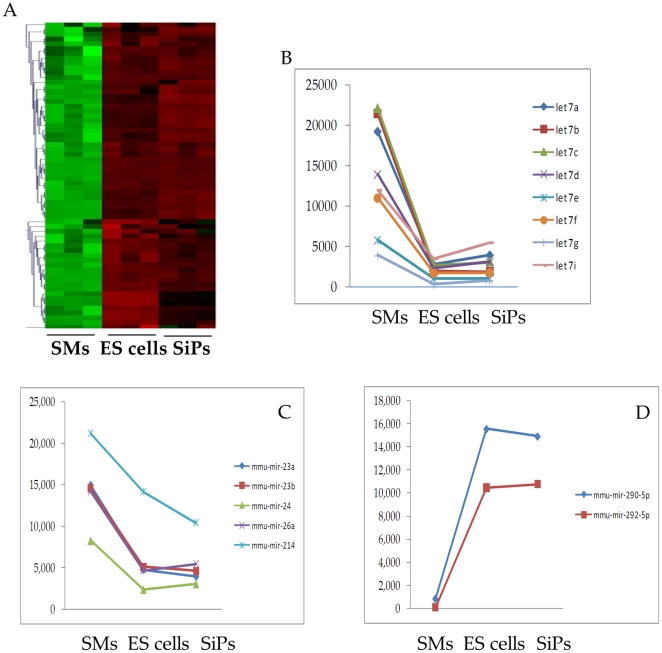
miR expression profile in SMs, SiPS and ES cells. (A) Heat map image of microarray showing miR expression profile in ES cells, and SiPS which was quite different from SMs. Red fluorescence indicates the increased expression and green fluorescence indicates decreased expression of miRs during reprogramming of SMs to SiPS. (B–D) Critical miRs known for reprogramming and differentiation of stem cells as observed in SMs, ES cells and SiPS.

Microarray analyses showed similar global gene expression profiles among SiPs and ES cells, which were very different from that of SMs (Figure 6A–D).

### *In vivo* transplantation of SiPS and SiPS-CPs

**Figure 7:**
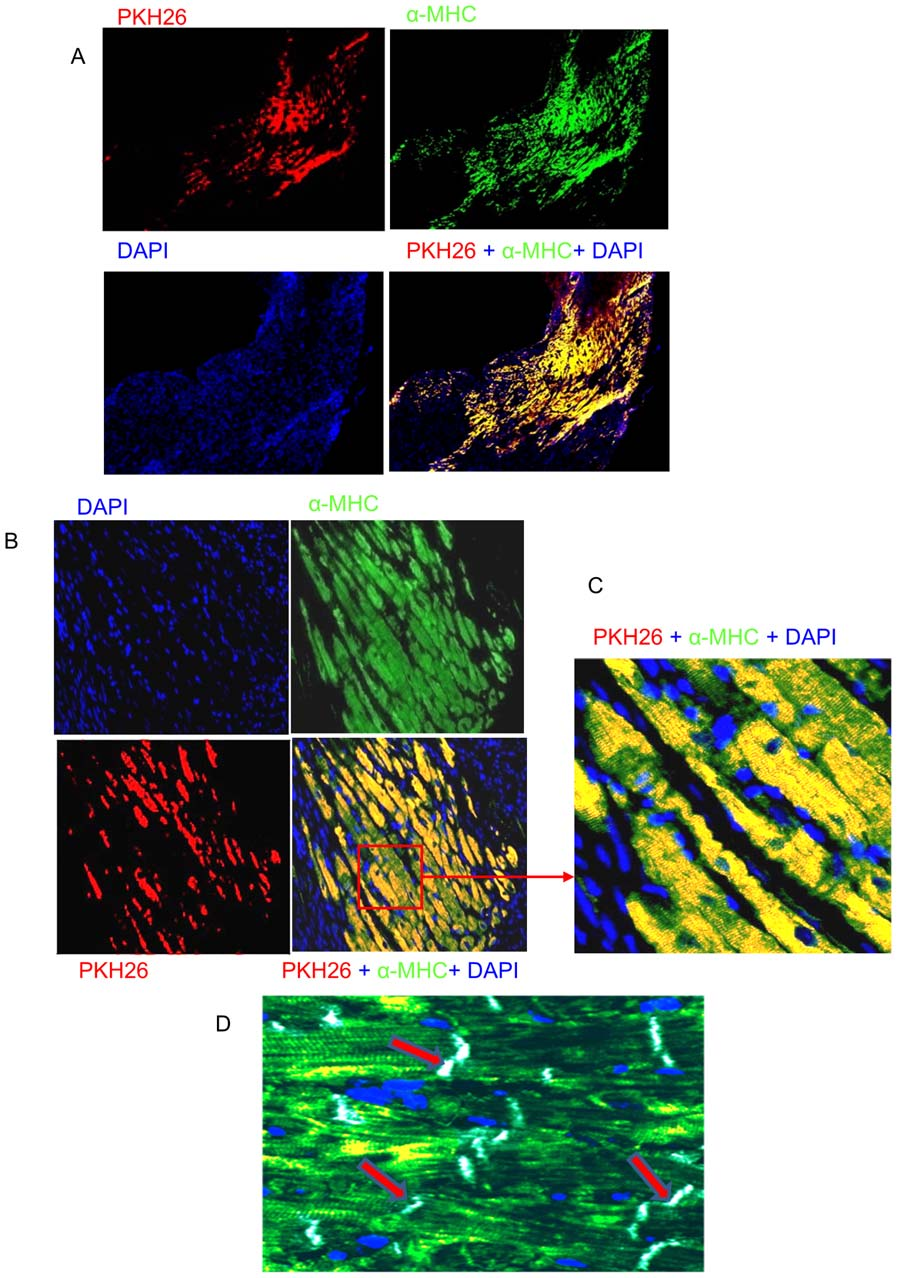
Histological evaluation of SiPS-CPs transplanted in the infarcted heart. (A–D) Microscopic images from recipient mouse hearts 4 weeks post-transplantation. The surviving 5 days EBs derived cardiac progenitors are identified by PKH26 (red fluorescence). The same area was stained for myosin heavy chain (green fluorescence) and the nuclei were stained with DAPI (blue fluorescence). The superimposed photo-images identified myosin as yellow in the merged images (A–D). The transplanted PKH26 labeled cardiac progenitors (yellow fluorescence) made contact with the host myocardium (green fluorescence) through gap junctions (white), a component of intercalated disks, as indicated by red arrows. (original magnifications: A = 10×, B = 20×, C & D = 40×).

We directly injected SiPS and 5 days beating EBS derived cardiac progenitors in the infarcted areas of left ventricle. We observed extensive survival, proliferation and differentiation of PKH26 labeled cardiac progenitors 4 weeks after transplantation in the infarcted region (Figure 7A–C). No tumor formation was observed in all the transplanted animals. Extensive myofibers were formed in the infarcted area. Co-localization of PKH26 (red fluorescence) with myosin heavy chain (green fluorescence) in the infarct and peri-infarct regions in the SiPS-CPs transplanted animal hearts indicated their myogenic differentiation (Figure 7A–D) Furthermore these transplanted SiPS-CPs made junctions with resident cardiomyocytes (Figure 7D).

### Left ventricular remodeling and cardiac function

**Figure 8:**
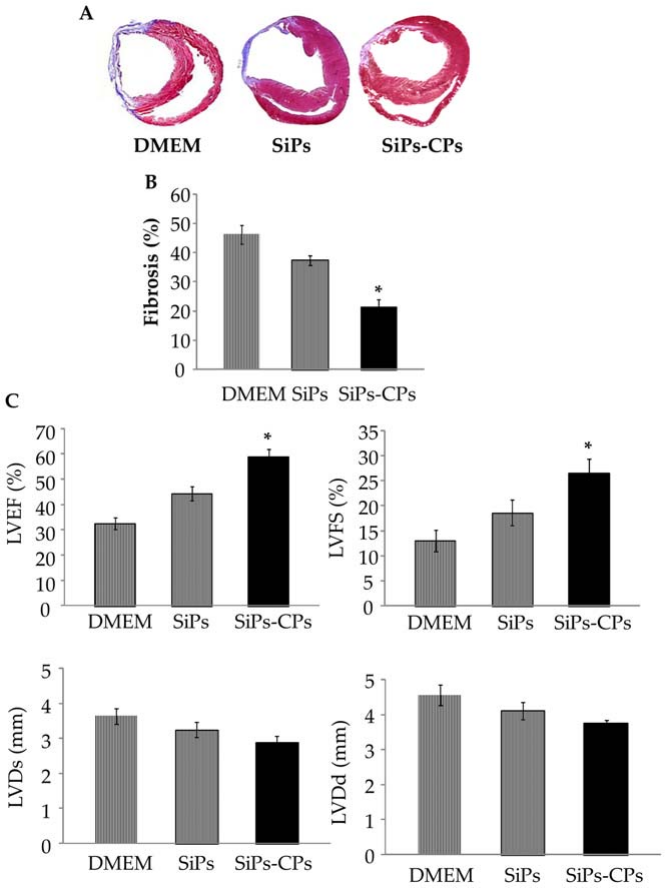
SiPS-CPs improved cardiac function and reduced infarction size 4 weeks post transplantation. (A) After 4 weeks of therapy, Masson's trichrome staining demonstrated reduced fibrosis (blue staining) in hearts treated with SiPS-CPs as compared with DMEM and SiPS. (B) Infarct size in various animal groups at 4 weeks after their respective treatment. (C) Assessment of cardiac function. Left ventricle ejection fraction (LVEF) and left ventricle fractional shortening (LVFS) showed significant improvement in the hearts treated with SiPS-CPs as compared to DMEM treated group and SiPS transplanted group. All values were expressed as mean± SEM. ^*^ *p*<0.05 *vs.* control group; (*n* = 6 per group).

Four weeks after respective treatments, we performed histochemical studies on at least two middle slices of the heart (n = 6 per group). Masson trichrome staining showed that SiPS-CPs treated animals had significantly reduced fibrosis as compared to DMEM injected controls (21.18±2.91% vs. 46.23±3.1%, *p*<0.01) and SiPS treated (37.35±1.7) (Figure 8A–B). The temporal changes in global heart function (Figure 8C) including left ventricle ejection fraction (LVEF) and left ventricle fractional shortening (LVFS) were measured as compared to the baseline values (n = 4; 78.1±2.8; 38.5±1.2% respectively). Transplantation of SiPS-CPs significantly improved LVEF and LVFS (58.9±3; 26.5±2.9) in comparison with the DMEM treated controls (32.61±2.35%; 13.06±2.2 *p*<0.05) and SiPS transplanted hearts (44.36±2.7; 18.66±2.52) respectively. The pathological remodeling of left ventricle in SiPS-CPs transplanted groups was also significantly reduced as shown by left ventricle chamber dimensions during systole (LVDs) and diastole (LVDd) (2.90±0.17 and 3.75±0.1) in comparison with DMEM (3.63±0.2 and 4.55±0.3) and SiPS treated group (3.25±0.21 and 4.11±0.25) respectively (Figure 8C).

## Discussion

Given the moral and ethical difficulties associated with the use of ES cells for transplantation, the novel approach to reprogram somatic cells to pluripotency has introduced a powerful research tool for creating substitute cells with greater therapeutic potentials. The use of retroviral vectors for induction of Yamanakas' quartet of transcription factors for iPS cell generation poses a serious risk of tumorgenesis due to their integration into the host genome [1]. This can be circumvented by the use of non integrating vectors either alone or in combination with small molecules. Small molecules have been used to modify chromatin structure and activate genes that are critical for restoring pluripotency. We hypothesized that somatic cells with endogenous expression of one or more stemness factors can make good candidates for reprogramming. In this regard, SMs which endogenously express three of the Yamanaks's quartet of stemness factors are therefore important candidates to generate iPS cells by induction of Oct3/4 expression which is lacking in SMs. We report here, for the first time, that small molecule RG108, an inhibitor of DNMT, was sufficient to reprogram mouse SMs to pluripotency with greater efficiency and convenience. The significant findings of this study are: (a) SMs can be reprogrammed to pluripotent status with higher efficiency and the reprogrammed SMs exhibited morphological, molecular, and functional properties similar to the ES cells. (b) Development and optimization of virus free strategy for generation of iPS cells with small molecule treatment of SMs which involved DNA methyltransferase inhibition. (c) Undifferentiated SiPS cells are tumorgenic in immunodeficient hosts (d) “Directed” spontaneous cardiac differentiation of SiPS which expressed cardiac-specific transcription factors and structural proteins and showed synchronous beating activity *in vitro*. (e) SiPS expressed ES cell like microRNA expression profile during reprogramming. (f) Pre-differentiation of SiPS into cardiac progenitor cells was ideal for tumor-free regeneration of infarcted myocardium.

Inclusion of small molecules in the reprogramming protocols is effective strategy to improve the efficiency of somatic cell reprogramming. However, our protocol which solely involved treatment of the cells with small molecules for induction of Oct3/4 for their efficient reprogramming to pluripotency is an important new development [16]–[18]. SMs possess inherent myogenic potential and are a good candidate for pluripotency due to intrinsic expression of three of the four pluripotency determinant transcription factors. Moreover, SMs have been extensively studied for myocardial repair in both experimental animal models and human patients [19]–[21]. Nevertheless, their lack of functional integration post-engraftment in the heart and the incidence of arrhythmia have limited their clinical applicability. It is highly likely that the reprogrammed SMs would provide a superior source of cardiac lineage cells for the repair of cardiovascular diseases.

*In vitro* reprogramming is a very slow and inefficient process and is associated with unforeseen risks such as subtle genetic and epigenetic abnormalities in iPS cells. Histone acetylation and methylation are important to the epigenetic status of the cells and regulate the accessibility of the transcription machinery to the DNA. Inhibition of histone deacetylase or DNA methyltransferase with the use of epigenetic modifiers such as valproic acid and azacytidine, alters chromatin remodeling and increases the efficiency of standardized reprogramming protocols [22], [11]. Various combinations of reprogramming factors with concomitant small molecule treatment have been used for reprogramming of somatic cells to yield better quality iPS cells with more efficiency [23], [24]. Consequently, it is highly desirable to identify new conditions/small molecules that can promote reprogramming and/or replace certain factors. In a recent study, SMs were reprogrammed using retroviral vectors which showed that Oct3/4 induction was associated with MYoD suppression [25]. This is in agreement with our findings that during reprogramming with RG108, SMs lost the myogenic specific genes. Our study is the first to show that somatic cells can be efficiently reprogrammed with single small molecule without genetic modification using exogenous stemness gene encoding viral vectors.

The present study highlights the unique advantage of complete chemical approach for reprogramming that may ultimately allow the generation of iPS cells in defined conditions without permanent genetic modification. Our data provide a solid foundation for developing regenerative medical therapies, including availability of alternate sources for autologous cardiac stem cells, and patient specific generation of iPS cell lines for the study of rare diseases. However, a number of issues still need to be addressed that include a complete understanding of expression of pluripotency genes in different somatic cells without non-viral approach and minimum transcriptional involvement with maximum reprogramming efficiency.

Besides improved efficiency of reprogramming, another significant finding of this study was spontaneous development of cardiac like cells *in vitro* which showed synchronous beating activity. RT-PCR and fluorescence immunostaining confirmed that these cells expressed cardiac-specific transcription factors and proteins. Although several studies have shown that iPS cells possess ES cell like characteristics, very few have shown their application for cardiac regeneration. The age of beating EBs is important for selection of myocytes for transplantation. We discovered that differentiating myocytes from older EBS derived cardiac progenitors were not as effective as the ones from 5 days old EBs (Data not shown). Therefore, we opted to transplant cardiac progenitors from 5 days beating EBs in this study. We not only observed extensive myogenesis in SiPS-CPs transplanted hearts but also these neomyocytes formed gap junctions with juxtaposed resident cardiomyocytes. With the precise removal of EBs having minimal contamination from undifferentiated SiPS for transplantation, no tumorgenesis was observed in the animal hearts treated with SiPS-CPs. Nevertheless, development of methods to enhance the purity of SiPS-CPs preparation for transplantation combined with our chemical method of reprogramming would be important in curtailing tumorgenic nature of iPS cells [26]–[27] with efficient cardiomyogenic differentiation potential.

We also observed that SiPS resembled ES cells in their miR expression profile thus suggesting their regulatory control over the fundamental processes including cell proliferation, cell differentiation, cell survival and cellular responses to the environment [28]–[29]. Significant changes were noted in the expression pattern of let-7 family, miR-200 and miR-290 family in SiPS in comparison with the native SMs. The let-7 family and miR-200 family were downregulated in SiPS as compared to SMs which was similar to ES cells indicated that SiPS were completely reprogrammed with small molecule treatment. Additionally, the importance of significant changes in expression of miRs including miR-21, miR-23 and miR-26 observed during the course of this study needs to be further investigated.

In summary, SMs with endogenous Sox2, Klf4, and cMyc are excellent candidates to generate iPS cells by upregulation of only Oct3/4 with the use of DNMT inhibitor. RG108 treatment of SMs not only upregulated Oct3/4 but also increased the expression of other already present factors. Reprogramming of SMs by RG108 is a simple, highly reproducible, safer and efficient technique more likely to regenerate patient specific cardiac progenitors for the treatment of cardiovascular disease. Future studies however would be required to determine the versatility of RG108 treatment and observe that our small molecule treatment approach would be efficient

## Supporting Information

Figure S1 **Quantitative RT-PCR analysis.** Relative mRNA expression levels of OCT4, SOX2, KLf4, cMyc, MyoD and PAX7 in SMs, ES cells and SiPS.
(DOCX)

Figure S2 **Quantitative RT-PCR analysis.** Relative mRNA expression levels of GATA4, Nkx2.5, Mef2C and αMHC in SMs, Heart, ES cells, 5-days EBs (EB) and SiPS.
(DOCX)

Table S1 **Sequences of the primers used during the studies.**
(DOCX)

Video S1 **Spontaneously beating SiPS derived cardiac progenitors in cell culture.** The EBs were cultured for 5 days in differentiation medium in the absence of LIF. Spontaneously beating regions in 5 days EBs in cell culture were observed which increased with the passage of time.
(AVI)

Text S1 **Supporting methods.**
(DOCX)

## References

[1] Takahashi K, Yamanaka S (2006). Induction of pluripotent stem cells from mouse embryonic and adult fibroblast cultures by defined factors.. Cell.

[2] Hanna J, Wernig M, Markoulaki S, Sun CW, Meissner A (2007). Treatment of sickle cell anemia mouse model with iPS cells generated from autologous skin.. Science.

[3] Hanna J, Markoulaki S, Schorderet P, Carey BW, Beard C (2008). Direct reprogramming of terminally differentiated mature B lymphocytes to pluripotency.. Cell.

[4] Wernig M, Meissner A, Cassady JP, Jaenisch R (2008). c-Myc is dispensable for direct reprogramming of mouse fibroblasts.. Cell Stem Cell.

[5] Stadtfeld M, Nagaya M, Utikal J, Weir G, Hochedlinger K (2008). Induced pluripotent stem cells generated without viral integration.. Science.

[6] Okita K, Nakagawa M, Hyenjong H, Ichisaka T, Yamanaka S (2008). Generation of mouse induced pluripotent stem cells without viral vectors.. Science.

[7] Kaji K, Norrby K, Paca A, Mileikovsky M, Mohseni P (2009). Virus-free induction of pluripotency and subsequent excision of reprogramming factors.. Nature.

[8] Woltjen K, Michael IP, Mohseni P, Desai R, Mileikovsky M (2009). piggyBac transposition reprograms fibroblasts to induced pluripotent stem cells.. Nature.

[9] Nakagawa M, Koyanagi M, Tanabe K, Takahashi K, Ichisaka T (2008). Generation of induced pluripotent stem cells without Myc from mouse and human fibroblasts.. Nat Biotechnol.

[10] Nichols J, Zevnik B, Anastassiadis K, Niwa H, Klewe-Nebenius D (1998). Formation of pluripotent stem cells in the mammalian embryo depends on the POU transcription factor Oct4.. Cell.

[11] Huangfu D, Osafune K, Maehr R, Guo W, Eijkelenboom A (2008). Induction of pluripotent stem cells from primary human fibroblasts with only Oct4 and Sox2.. Nat Biotechnol.

[12] Shi Y, Desponts C, Do JT, Hahm HS, Schöler HR (2008). Induction of pluripotent stem cells from mouse embryonic fibroblasts by Oct4 and Klf4 with small-molecule compounds.. Cell Stem Cell.

[13] Huangfu D, Maehr R, Guo W, Eijkelenboom A, Snitow M (2008). Induction of pluripotent stem cells by defined factors is greatly improved by small-molecule compounds.. Nat Biotechnol.

[14] Brueckner B, Garcia Boy R, Siedlecki P, Musch T, Kliem HC (2005). Epigenetic reactivation of tumor suppressor genes by a novel small-molecule inhibitor of human DNA methyltransferases.. Cancer Res.

[15] Wang Y, Haider HK, Ahmad N, Xu M, Ge R (2006). Combining pharmacological mobilization with intramyocardial delivery of bone marrow cells over-expressing VEGF is more effective for cardiac repair.. J Mol Cell Cardiol.

[16] Dann CT, Alvarado AL, Molyneux LA, Denard BS, Garbers DL (2008). Spermatogonial stem cell self-renewal requires OCT4, a factor downregulated during retinoic acid-induced differentiation.. Stem Cells.

[17] Pardo M, Lang B, Yu L, Prosser H, Bradley A (2010). An expanded Oct4 interaction network: implications for stem cell biology, development, and disease.. Cell Stem Cell.

[18] Buehr M, Nichols J, Stenhouse F, Mountford P, Greenhalgh CJ (2003). Rapid loss of Oct-4 and pluripotency in cultured rodent blastocysts and derivative cell lines.. Biol Reprod.

[19] Haider H, Ye L, Jiang S, Ge R, Law PK (2004). Angiomyogenesis for cardiac repair using human myoblasts as carriers of human vascular endothelial growth factor.. J Mol Med.

[20] Jain M, DerSimonian H, Brenner DA, Ngoy S, Teller P (2001). Cell therapy attenuates deleterious ventricular remodeling and improves cardiac performance after myocardial infarction.. Circulation.

[21] Menasche P, Hagege AA, Scorsin M, Pouzet B, Desnos M (2001). Myoblast transplantation for heart failure.. Lancet.

[22] Mikkelsen TS, Hanna J, Zhang X, Ku M, Wernig M (2008). Dissecting direct reprogramming through integrative genomic analysis.. Nature.

[23] Han J, Sachdev PS, Sidhu KS (2010). A combined epigenetic and non-genetic approach for reprogramming human somatic cells.. PLoS One.

[24] Han JW, Yoon YS (2011). Induced Pluripotent Stem Cells: Emerging Techniques for Nuclear Reprogramming.. Antioxid Redox Signal.

[25] Watanabe S, Hirai H, Asakura Y, Tastad C, Verma M (2011). Myod Gene Suppression by Oct4 is required for reprogramming in myoblasts to produce induced pluripotent stem cells.. Stem Cells.

[26] Ahmed RP, Ashraf M, Buccini S, Jiang S, Haider HK (2011). Cardiac tumorigenic potential of induced pluripotent stem cells in an immunocompetent host with myocardial infarction.. Regen Med.

[27] Ahmed RP, Haider HK, Buccini S, Li L, Jiang S (2011). Reprogramming of skeletal myoblasts for induction of pluripotency for tumor-free cardiomyogenesis in the infarcted heart.. Cir Res.

[28] Stefani G, Slack F (2006). MicroRNAs in search of a target.. Cold Spring Harb Symp Quant Biol.

[29] Leung AK, Sharp PA (2010). MicroRNA functions in stress responses.. Mol Cell.

