# Supplementary material for: Efficient Non-Viral Reprogramming of Myoblasts to Stemness with a Single Small Molecule to Generate Cardiac Progenitor Cells
Source: PLoS One. 2011 Aug 17;6(8):e23667. doi: 10.1371/journal.pone.0023667 (PMC3157438; doi:10.1371/journal.pone.0023667)
Supplement: Figure S1 — Quantitative RT-PCR analysis. Relative mRNA expression levels of OCT4, SOX2, KLf4, cMyc, MyoD and PAX7 in SMs, ES cells and SiPS. (DOCX) [file pone.0023667.s001.docx]

**Pasha *et al*. 2011 Supporting Figure-S1**
