# Supplementary material for: Efficient Non-Viral Reprogramming of Myoblasts to Stemness with a Single Small Molecule to Generate Cardiac Progenitor Cells
Source: PLoS One. 2011 Aug 17;6(8):e23667. doi: 10.1371/journal.pone.0023667 (PMC3157438; doi:10.1371/journal.pone.0023667)
Supplement: Table S1 — Sequences of the primers used during the studies. (DOCX) [file pone.0023667.s003.docx]

**Supplemental Table-SI:** Sequences of the primers used during the studies

Gene Sequence Product size

Oct4 for5'-cacgagtggaaagcaactca

rev5’-agatggtggtctgg ctgaac (290bp)

Sox2 for5'-cacaactcggagatcagcaa,

rev5'-ctccgggaagcgtgtactta (190bp)

Klf for5'-gttggcgtgaggaactctct

rev5'-gtgggttagcgagttggaaa (212bp)

cMyc for5'-gcccagtgaggatatctggarev

rev5'-atcgcagatgaagctctggt (226bp)

Nanog for5’-gc accaactcaacttctgagcrev

rev5’-ctcgagagtagccaccatatc (286bp)

Mef2c for5’-agcaagaatacgatgccatc-3

rev5’-gaaggggtggtggtacggtc-3 (407bp)

Gata4 for5'- ctgtcatctcactatgggca

rev 5'- ccaagtccgagcaggaattt (255bp)

TERT for5’-ctgcgtgtgcgtgctctggac

rev5’-cacctcagcaaacagcttgttctc (498bp)

Nestin for5’-atacaggactctgctggagg

rev5’-aggacaccagtagaactggg (410bp)

Rex1 for5’-ggccagtccagaataccaga

rev5’gaactcgcttccagaacctg (232bp)

Nkx2.5 for5’-tctccgatccatcccactttattg

rev5’-gcgttagcgcactcactttaatg (222bp)

α-Mhc for5’-accgtggactacaacat

rev5’-ctttcgctcgttggga (287bp)

TnT for5’-gcggaagagtgggaagagaca

rev5’-ccacagctccttggccttct (127 bp)

gapdh for 5’-tggccttccgtgttcctacc

rev5’-tgtaggccatgaggtccaccac (300bp)

Mef2c for 5-agcaagaatacgatgccatc-3

rev 5-gaaggggtggtggtacggtc-3 (407 bp)

MyoD for 5'-cacgactgctttcttcaccactcc

rev 5'- cgcaggtctggtgagtcgaaacac (298 bp)

Pax 7 for 5'-gcttggtggggtcttcatcaacgg

rev 5'-ctgagcactcggctaatcgaactc (357bp)
