# Supplementary material for: Efficient Non-Viral Reprogramming of Myoblasts to Stemness with a Single Small Molecule to Generate Cardiac Progenitor Cells
Source: PLoS One. 2011 Aug 17;6(8):e23667. doi: 10.1371/journal.pone.0023667 (PMC3157438; doi:10.1371/journal.pone.0023667)
Supplement: Text S1 — Supporting methods. (DOCX) [file pone.0023667.s005.docx]

**Supporting Information**

**Supporting Methods**

**Isolation of mouse SMs**

For our animal experiments, we used the Oct4/GFP transgenic mouse strain (Jackson laboratories, Maine, USA) with GFP-tagged to the endogenous Oct3/4 gene which helped to monitor the reprogramming process as the reprogrammed cells showed green fluorescence. For SMs isolation, young (4-6 weeks old) male Oct3/4-GFP transgenic mice were sacrificed and skeletal muscle samples from the hind-limb were harvested immediately and kept in ice cold basal Medium -199 (M-199; Mediatech, VA, USA) before digestion. The muscle samples were minced and enzymatically digested using Collagenase-1/Dispase (Roche Diagnostics) for 30 minutes at 37°C. The tissue slurry was washed with low serum containing M-199 (2% fetal calf serum), centrifuged at 1000rpm for 5-minutes and filtered through 90nm nylon mesh to remove any tissue debris. The muscle extract was pre-plated three times at an interval of 1 hour each and twice additionally at 8 and 16 hours to remove the debris and contaminating cell populations. After the last pre-plating, 0.1m mmol/L 5-bromodeoxyuridine (BD Pharmingen, CA, USA) was added to the cell culture for 3 days to inhibit fibroblast growth followed by 3 days of treatment with 15ng/mL basic fibroblast growth factor (bFGF) (Sigma Aldrich, MO, USA). The cells were later propagated in M-199 supplemented with 20% fetal bovine serum (FBS) at 37°C and 5% CO2 atmosphere. The purity and uniformity of myoblast preparation was assessed by immunostaining for desmin expression for all the batches of SMs used in the study. At least two microscopic slides grown with SMs were immunostained for each batch of cells. The percentage of desmin positive myoblast was calculated from the ratio between the stained and the unstained cells by counting the 24 various microscopic fields in all the myoblast batches used in the studies. The purified SMs were repeatedly passaged at low cell density at regular time intervals to prevent their premature differentiation *in vitro*.

**Maintenance of mouse SiPS**

We maintained SiPS clone ZP1 on mitomycin C-treated MEFs dishes in Knock out Dulbecco's Modified Eagle's Medium (knock out-DMEM, Invitrogen, CA, USA) supplemented with 20% Knockout Serum Replacement (KSR; Invitrogen, USA), 0.1 mM MEM Non-Essential Amino Acids solution (Invitrogen, CA, USA), 0.2 mM L- glutamine (Invitrogen, USA), 0.1 mM β- mercaptoethanol (Invitrogen, CA, USA) and 1 000 U/ml LIF (Millipore) 0.5% penicillin and streptomycin. The colonies thus generated were detached regularly at an interval of 3-4 days with 0.2% collagenase-1V (Invitrogen, CA,

USA) dissociated into single cell suspension with 0.025% trypsin (Sigma Aldrich, MO, USA) and re-plated onto MEFs for propagation.

**RT-PCR and Quantitative RT-PCR**

Total RNA from SMs, ES cells, SiPS Cells, 5 days EBs and mouse heart tissue was isolated using RNeasy mini kit (Qiagen, Maryland, USA) and Omniscript Reverse Transcription kit (Qiagen, Maryland, USA) was used for the respective cDNA synthesis per manufacturer’s instructions. The primers used are given in Supplementary information, Table-S1. For PCR amplification, 1μg of the cDNA from the reverse transcription reaction was added to PCR mix containing the suggested quantity of the PCR buffer, Q solution, dNTP mix, reverse and forward primers, Taq DNA polymerase and distilled water. PCR conditions included initial denaturation at 95°C for 4 minutes, 32 cycles of denaturation at 95°C for 1 minute, annealing at 55°C for 1 minute, extension at 72°C for 1 minute and final extension at 72°C for 7 minutes. The PCR products were separated on 1.5 % agarose gel, stained with ethidium bromide and visualized and photographed on a UV transluminator (Bio-Rad, USA).

**Alkaline phosphatase staining and immunocytochemistry**

Alkaline phosphatase staining was performed using Alkaline Phosphatase Detection kit (Millipore) per manufacturer's instructions. For immunocytochemistry, SMs or differentiated colonies of SiPS were immunostained with respective specific primary antibodies (anti desmin, anti- SSEA1, anti-Oct3/4, anti-Sox2, anti α-fetoprotein, anti β-tubulin antibodies, all at 1:100 concentration; Cell Signaling, Danvers, USA). Five days beating EBs were mechanically dissected and seeded on 0.1 % gelatin coated chambered slides for immunostaining. The cells were fixed with PBS containing 4% paraformaldehyde for 10 minutes at room temperature. After washing with PBS, the cells were blocked for 45 minutes at room temperature by CAS block (Invitrogen, CA, USA) and were immunostained with antigen specific primary antibodies GATA4,

Nkx2.5, Mef2c, MHC, Connexin-43, N-cadherin (Santa Cruz, CA, USA). The primary antibody-antigen reaction was detected with fluorescently conjugated specific secondary antibodies. Nuclei were stained with 5 μg/ml 4′6-diamidino-2- phenyl indole (DAPI; Invitrogen, CA, USA) staining. Fluorescence signals were observed and photographed using fluorescence microscopy (Oympus; Tokyo, Japan).

**Ultra-structural studies of EBs derived cardiomyocytes**

Cardiac progenitors derived from 5-day beating EBs were examined by transmission electron microscopy. The beating cells were fixed in 2% glutaraldehyde made in 0.1 M cacodylate buffer (pH 7.4) at 4⁰C for 42 hours. The cell pellets were then post fixed in 1% OsO4 in the same buffer followed by uranyl acetate staining for 30 minutes at room temperature. The cell pellet was then dehydrated in graded series of 50%, 70%, 90% and 100% ethanol and embedded in Epon 812. Thin sections (~60nm) were cut with ultra microtome, placed on a grid and after staining with lead citrate, were viewed in transmission electron microscope (JEOL Ltd, USA).

**Micro RNA microarray Analysis and Karyotyping**

Micro RNA expression profiling was performed using a microarray service provider (LC Sciences). A total of 4-8 μg RNA sample was prepared from SMs, mouse ESCs and SiPS, which was size fractionated using YM-100 Microcon centrifugal filter (Millipore). The small RNAs (<300 nt) isolated were 3’-extended with a poly (A) tail using poly (A) polymerase. An oligonucleotide tag was then ligated to the poly (A) tail for later fluorescent dye staining; two different tags were used for the two RNA samples in dual-sample experiments. Hybridization was performed overnight on a Paraflo microfluidic chip using a micro-circulation pump (Atactic Technologies). On the microfluidic chip, each detection probe consisted of a chemically modified nucleotide coding segment complementary to target microRNA (from miRBase, http://microrna. sanger.ac.uk/ sequences) or other RNA (control or customer defined sequences) and a spacer segment of polyethylene glycol to extend the coding segment away from the substrate. The detection probes were made by *in situ* synthesis using PGR (photogenerated reagent) chemistry. The hybridization melting temperatures were balanced by chemical modifications of the detection probes. Hybridization used 100 μL 6xSSPE buffer (0.90 M NaCl, 60 mM Na2HPO4, 6 mM EDTA, pH 6.8) containing 25% formamide at 34 °C. After RNA hybridization, tag-conjugating Cy3 and Cy5 dyes were circulated through the microfluidic chip for dye staining. Fluorescence images were collected using a laser scanner (GenePix 4000B, Molecular Device) and digitized using Array-Pro image analysis software (Media Cybernetics). Data were analyzed by first subtracting the background and then normalizing the signals using a LOWESS filter (Locally-weighted Regression). For two color experiments, the ratio of the two sets of detected signals (log2 transformed, balanced) and p-values of the *t*-test were calculated; differentially detected signals were those with less than 0.01 p-values. Karyotyping was determined at molecular cytogenetics facility of Ohio State University, OH.

**Teratoma formation**

Immunodeficient nude mice were purchased from Jackson Laboratories, USA. Undifferentiated SiPS (1x105 cells) were injected subcutaneously into 2-3 month old nude immunodeficient mice. Two weeks after injection, teratomas were fixed with 10 % formalin and stained with hematoxylin and eosin (HE) for three germ layer differentiation evaluation.

**Histochemical and Immunohistochemical studies**

For measurement of infarction size and area of fibrosis, the heart was arrested in diastole by intravenous injection of cadmium chloride and fixed using formalin. The heart was then excised, cut transversely, and embedded in paraffin. Histological sections of 6μm thickness were cut and used for hematoxylin-eosin and Masson’s trichrome staining for visualization of muscle architecture and thickness of the LV wall. Infarct size was defined as the sum of the epicardial and endocardial infarct circumference divided by the sum of the total LV epicardial and endocardial circumferences using computer-based planimetry with Image-J analysis software (version 1.6065; NIH). For myogenic differentiation of the transplanted cells, paraffin sections (6μm thickness) were immunostained using specific antibodies for α-myosin heavy chain (α-MHC) and detected with FITC-labeled secondary antibody (1:1000 dilution).
